# Targeting *FLT3* mutations in AML: review of current knowledge and evidence

**DOI:** 10.1038/s41375-018-0357-9

**Published:** 2019-01-16

**Authors:** Naval Daver, Richard F. Schlenk, Nigel H. Russell, Mark J. Levis

**Affiliations:** 1 Department of Leukemia, The University of Texas MD Anderson Cancer Center, Houston, TX USA; 2 National Center of Tumor Diseases, German Cancer Research Center, Heidelberg, Germany; 3 Centre for Clinical Haematology, Nottingham University Hospitals NHS Trust, Nottingham, UK; 4 Sidney Kimmel Comprehensive Cancer Center, Johns Hopkins University, Baltimore, MD USA

**Keywords:** Acute myeloid leukaemia, Drug development

## Abstract

Genomic investigations of acute myeloid leukemia (AML) have demonstrated that several genes are recurrently mutated, leading to new genomic classifications, predictive biomarkers, and new therapeutic targets. Mutations of the FMS-like tyrosine kinase 3 (*FLT3*) gene occur in approximately 30% of all AML cases, with the internal tandem duplication (ITD) representing the most common type of *FLT3* mutation (*FLT3*-ITD; approximately 25% of all AML cases). *FLT3*-ITD is a common driver mutation that presents with a high leukemic burden and confers a poor prognosis in patients with AML. The prognostic value of a *FLT3* mutation in the tyrosine kinase domain (*FLT3*-TKD), which has a lower incidence in AML (approximately 7–10% of all cases), is uncertain. Accumulating evidence demonstrates that *FLT3* mutational status evolves throughout the disease continuum. This so-called clonal evolution, together with the identification of *FLT3*-ITD as a negative prognostic marker, serves to highlight the importance of *FLT3*-ITD testing at diagnosis and again at relapse. Earlier identification of *FLT3* mutations will help provide a better understanding of the patient’s disease and enable targeted treatment that may help patients achieve longer and more durable remissions. First-generation FLT3 inhibitors developed for clinical use are broad-spectrum, multikinase inhibitors; however, next-generation FLT3 inhibitors are more specific, more potent, and have fewer toxicities associated with off-target effects. Primary and secondary acquired resistance to FLT3 inhibitors remains a challenge and provides a rationale for combining FLT3 inhibitors with other therapies, both conventional and investigational. This review focuses on the pathological and prognostic role of *FLT3* mutations in AML, clinical classification of the disease, recent progress with next-generation FLT3 inhibitors, and mechanisms of resistance to FLT3 inhibitors.

## AML genetic landscape: risk categorization and recommendations for *FLT3* testing

Acute myeloid leukemia (AML) is a malignancy of proliferative, clonal, abnormally, or poorly differentiated cells of the hematopoietic system, characterized by clonal evolution and genetic heterogeneity [1, 2]. Genetic alterations are recurrent and include amplifications, deletions, rearrangements, and point mutations [2, 3]. Because cytogenetic profiles are important prognostic indicators of clinical outcomes, characterizing chromosomal abnormalities in AML helps stratify patients according to risk and guide therapeutic decisions.

Prognostic risk is defined at diagnosis based on the presence of certain cytogenetic and molecular aberrations [4–7]. Guidelines for AML classification and risk stratification have been established by several organizations, including the World Health Organization (WHO), National Comprehensive Cancer Network (NCCN), and European LeukemiaNet (ELN) [4, 5]. Although the WHO lists FMS-like tyrosine kinase 3 internal tandem duplication (*FLT3*-ITD) as a molecular genetic alteration significantly affecting the clinical outcome in patients with AML in specific cytogenetic subgroups [7], it does not group *FLT3* mutations into a single category but rather divides them into many subgroups. Thus, the focus of this review will be on the latter two sets of guidelines, NCCN and ELN.

The NCCN and ELN guidelines (Tables 1 and 2) stratify patients into three risk groups: favorable, intermediate, and poor/adverse. The NCCN Clinical Practice Guidelines in Oncology classify patients with AML with normal cytogenetics harboring the *FLT3*-ITD or *TP53* mutations as poor risk. Additionally, because *FLT3*-ITD mutations are considered to confer a significantly poor outcome, these guidelines suggest that patients with these mutations should be considered for clinical trials if possible [5]. As for the ELN guidelines, these have recently undergone substantial revisions, the most significant of which includes categorization into only three risk groups based on genetics vs. four groups in the earlier classification. Importantly, with these revisions, AML with *FLT3*-ITD^high^ allelic ratio (>0.5) in the absence of mutant nucleophosmin (*NPM1*) was moved into the high-risk group. Other significant changes include the addition of a new response category, “complete remission with no evidence of measurable residual disease,” and the inclusion of additional gene mutations (*RUNX1*, *ASXL1*, and *TP53*) defining high risk. A new provisional response category, “progressive disease,” was also added, particularly for use in clinical trials. The response category “stable disease,” with the period of stable disease considered to be at least 3 months [4], was also added for clinical trial use.

**Table 1:** NCCN 2017 AML risk stratification based on validated cytogenetics and molecular abnormalities [5]

Risk status	Cytogenetics	Molecular abnormalities
Favorable risk	Core binding factor: inv(16) or t(16;16) or t(8;21) or t(15;17)	Normal cytogenetics: *NPM1* mutation in the absence of *FLT3*-ITD or isolated biallelic (double) *CEBPA* mutation
Intermediate risk	Normal cytogenetics: +8 alone t(9;11) Other nondefined	Core binding factor with *KIT* mutation
Poor risk	Complex (≥3 clonal chromosomal abnormalities): Monosomal karyotype −5, 5q−, −7, 7q− 11q23 – non t(9;11) inv(3), t(3;3) t(6;9) t(9;22)	Normal cytogenetics: With *FLT3*-ITD mutation or *TP53* mutation

*AML* acute myeloid leukemia, *CEBPA* CCAAT/enhancer-binding protein alpha, *FLT3* FMS-like tyrosine kinase 3, *ITD* internal tandem duplication, *NCCN* National Comprehensive Cancer Network, *NPM1* nucleophosmin

**Table 2:** ELN 2017 AML risk stratification by genetics [4]

Risk status	Genetic abnormality
Favorable	t(8;21)(q22;q22.1); *RUNX1*-*RUNX1T1* inv(16)(p13.1;q22) or t(16;16)(p13.1;q22); *CBFB-MYH11* Mutated *NPM1* without *FLT3*-ITD or with *FLT3*-ITD^low,a^ Biallelic mutated *CEBPA*
Intermediate	Mutated *NPM1* and *FLT3*-ITD^high,a^ Wild-type *NPM1* without *FLT3*-ITD or with *FLT3*-ITD^low,a^ (without adverse-risk genetic lesions) t(9;11)(p21.3;q23.3); *MLLT3-KMT2A*^b^ Cytogenetic abnormalities not classified as favorable or adverse
Adverse	t(6;9)(p23;q34.1); *DEK-NUP214* t(v;11q23.3); *KMT2A* rearranged t(9;22)(q34.1;q11.2); *BCR-ABL1* inv(3)(q21.3;q26.2) or t(3;3)(q21.3;q26.2); *GATA2*, *MECOM(EVI1)* −5 or del(5q); −7; −17/abn(17p) Complex karyotype,^c^ monosomal karyotype^d^ Wild-type *NPM1* and *FLT3*-ITD^high,a^ Mutated *RUNX1*^e^ Mutated *ASXL1*^e^ Mutated *TP53*^f^

*AML* acute myeloid leukemia, *CEBPA* CCAAT/enhancer-binding protein alpha, *ELN* European LeukemiaNet, *FLT3* FMS-like tyrosine kinase 3, *ITD* internal tandem duplication, *NPM1* nucleophosmin, *WT* wild-type

Frequencies, response rates, and outcome measures should be reported by risk category, and, if sufficient numbers are available, by specific genetic lesions indicated

Prognostic impact of a marker is treatment dependent and may change with new therapies

^a^Low, low allelic ratio (<0.5); high, high allelic ratio (≥0.5). Semiquantitative assessment of *FLT3*-ITD allelic ratio (using DNA fragment analysis) is determined as the ratio of the area under the curve “*FLT3*-ITD” divided by the area under the curve “*FLT3*-wild-type.” Recent studies indicate that AML with *NPM1* mutation and *FLT3*-ITD low allelic ratio may have a more favorable prognosis and that patients should not routinely be assigned to allogeneic hematopoietic stem cell transplant

^b^The presence of t(9;11)(p21.3;q23.3) takes precedence over rare, concurrent adverse-risk gene mutations

^c^Three or more unrelated chromosomal abnormalities in the absence of one of the World Health Organization–designated recurring translocations or inversions, ie, t(8;21), inv(16) or t(16;16), t(9;11), t(v;11)(v;q23.3), t(6;9), inv(3) or t(3;3), AML with *BCR-ABL1*

^d^Defined by the presence of one single monosomy (excluding loss of X or Y) in association with ≥1 additional monosomy or structural chromosomal abnormality (excluding core-binding factor AML)

^e^These markers should not be used as an adverse prognostic marker if they co-occur with favorable-risk AML subtypes

^f^*TP53* mutations are significantly associated with AML with complex and monosomal karyotypes

Both the NCCN and ELN guidelines recommend the inclusion of *FLT3* genetic testing in the diagnostic workup. More specifically, the NCCN guidelines recommend that *FLT3* testing be performed at diagnosis in all patients with AML, in parallel with cytogenetic testing, to identify those who may benefit from targeted treatment options [5]. ELN recommends that, along with *FLT3*-ITD screening, mutant-to-wild-type allelic ratio and tyrosine kinase domain (TKD) mutations at codons D835 and I836 should be assessed.

The risk classification for *NPM1* and *FLT3*-ITD genotypes in the 2017 ELN recommendations is presented in Table 2 [4] and was validated in a large retrospective analysis in patients with newly diagnosed AML and intermediate-risk cytogenetic abnormalities or normal karyotype [8]. A challenge with the ELN risk stratification is that it relies on *FLT3*-ITD allelic ratio data, which has yet to become part of the standard testing in clinical practice and is often unavailable to treating physicians. Additionally, while higher *FLT3*-ITD allelic ratios have been recognized for their association with poorer clinical outcomes [4], there is currently no internationally standardized methodology for determining these allelic ratios [9], further confounding the issue.

The observation that *FLT3* mutations can evolve from diagnosis to relapse suggests that testing for *FLT3*-ITD mutations may be necessary at multiple time points throughout a patient’s disease course to help guide the most appropriate therapeutic decisions. Because both the NCCN and ELN 2017 guidelines support prompt, comprehensive *FLT3* testing for all patients with AML [4, 5], use of a rapid *FLT3*-ITD diagnostic assay has the potential to improve patient care by identifying patients with AML with a poor prognosis and allowing early intervention with *FLT3*-ITD targeted therapies [9–11]. However, there are several challenges. For example, interpretation of test results may be difficult due to variability in diagnostic accuracy, sensitivity, and qualitative vs. quantitative readouts of different *FLT3* assays. Another source of variability stems from the timing of the testing and how patients are subsequently managed based on the physician’s interpretation of the assay results. Additionally, *FLT3*-ITD allelic ratio has been used more often for research purposes than in real-world practice; as such, the clinical impact of *FLT3*-ITD allelic ratio has thus far been assessed retrospectively in patient data sets but not confirmed prospectively. Lastly, the lack of standardized laboratory reference values for *FLT3*-ITD allelic ratio is a further limitation. These observations highlight an acute need for an international standard for *FLT3* mutational testing (Table 3), reporting, and interpretation.

**Table 3:** Comparison of *FLT3* testing methods [98]

*FLT3* testing technique	Specificity for *FLT3* mutations	Sensitivity^a^	Turnaround time
Fluorescence-labeled PCR	Highly specific (>99%); detects mutations only within amplified region	5%	3 days
Whole-genome sequencing	Unbiased approach; detects *FLT3*-ITD and other *FLT3* mutations	>20%	7–12 days
Whole-exome sequencing	Unbiased approach; detects *FLT3*-ITD and other *FLT3* mutations	>5%	Not reported; faster than whole-genome sequencing
Multiplex-targeted NGS	Unbiased approach; 99–100% detection of *FLT3* mutations	1–2%	3–20 days
Karyogene	Highly specific (100%); samples are enriched for *FLT3* exons	>5%	<14 days^b^
PCR based	Detects *FLT3*-ITD and *FLT3*-TKD mutations	1%	7–10 days

*FLT3* FMS-like tyrosine kinase 3, *ITD* internal tandem duplication, *NGS* next-generation sequencing, *PCR* polymerase chain reaction, *TKD* tyrosine kinase domain

^a^Detection of mutant allele variants per fraction of total cells

^b^For samples run once weekly; turnaround time can be <10 days for samples run twice weekly

Although routine testing for *FLT3* mutations in patients with cytogenetically normal AML has been recommended by the ELN since 2010 [10], *FLT3* testing is not always performed. The significantly higher rates of *FLT3* testing at academic centers than at community sites suggests that there is a lack of awareness about the therapeutic implications of molecular testing in the community setting [12]. To validate this hypothesis, a large survey of physicians from the United States and Europe was conducted to assess their current practices in ordering tests for the diagnosis of acute leukemia. Only 51% of respondents indicated that they tested for *FLT3*-ITD in new AML referrals. Flow cytometry and karyotyping, on the other hand, were found to be routinely performed for the diagnosis of acute leukemia [13]. With increasing recognition of the importance of routine testing for *FLT3* mutations in AML and the availability of FLT3 inhibitors, the frequency of *FLT3* testing will likely increase in the future.

## *FLT3* mutations in AML

FLT3 is a transmembrane ligand-activated receptor tyrosine kinase that is normally expressed by hematopoietic stem or progenitor cells and plays an important role in the early stages of both myeloid and lymphoid lineage development [14]. An extracellular ligand (FLT3 ligand) binds and activates FLT3, promoting cell survival, proliferation, and differentiation through various signaling pathways, including PI3K, RAS, and STAT5 [14]. Mutations of *FLT3* are found in approximately 30% of newly diagnosed AML cases and occur as either ITDs (≈ 25%) or point mutations in the TKD (7–10%) [5, 9, 15, 16]. *FLT3*-ITD occurs in the form of a replicated sequence in the juxtamembrane domain and/or TKD1 of the FLT3 receptor and varies in location and length within these domains. Both *FLT3*-ITD and *FLT3*-TKD mutations constitutively activate FLT3 kinase activity, resulting in proliferation and survival of AML [14].

*FLT3*-ITD^high^ is a driver mutation that presents with a high leukemic burden, confers a poor prognosis, and has a significant negative impact on the management of patients with AML [1, 6, 9, 17–19]. Initial evidence of *FLT3*-ITD as a driver mutation came from comparative studies assessing the presence of this mutation in bone marrow (BM) samples at diagnosis and subsequently at relapse [20, 21]. A significant increase in the *FLT3*-ITD allelic ratio in relapsed AML indicates that a *FLT3*-ITD-mutated subclone present at diagnosis may possess a growth advantage and, through clonal expansion, become the dominant clone at relapse. Therefore, the *FLT3*-ITD mutation directly or indirectly confers a selective advantage to a clone in its microenvironment. Indeed, about 75% of patients with *FLT3*-ITD–mutated AML at diagnosis continue to have the ITD mutation at relapse [22], suggesting that *FLT3*-ITD may function as the driver mutation responsible for progressing the disease into overt leukemia. Additionally, more recent evidence from a large study of the mutational landscape in adult AML identified *FLT3* as one of the most commonly occurring mutations; specifically, *FLT3*-ITD was found to be one of the three most common drivers in patients with AML with intermediate-risk karyotypes (based on Medical Research Council classification) [23, 24], providing further support of its role as a driver mutation. More definitive evidence was observed with the first clinical use of potent FLT3 inhibitors, which resulted in the emergence of resistance-conferring point mutations [25], thus highlighting the function of *FLT3*-ITD mutations as drivers. In particular, the next-generation, highly specific FLT3 inhibitors—including gilteritinib, crenolanib, and quizartinib—demonstrated high single-agent activity, confirming the therapeutic potential of this approach [26–30]. Conversely, the first-generation multitargeted tyrosine kinase inhibitors (TKIs) that also inhibit FLT3 (e.g., midostaurin and sorafenib) initially demonstrated only modest single-agent activity in relapsed *FLT3*-ITD-mutated AML [31, 32], albeit with improved response rates and survival when combined with chemotherapy [33, 34].

## Prognostic impact of *FLT3* mutations in newly diagnosed AML

Patients with *FLT3*-ITD mutations tend to have a particularly unfavorable prognosis, with an increased risk of relapse and shorter overall survival (OS) compared with patients without the mutation [35, 36]. A recent meta-analysis demonstrated that the presence of *FLT3*-ITD is associated with a poor prognosis in terms of OS and relapse-free survival (RFS; hazard ratios of 1.86 and 1.75, respectively) [37]. In contrast, the prognostic impact of *FLT3*-TKD mutations is not as well defined. For example, several studies have found weak associations between clinical outcomes and the presence of *FLT3*-TKD mutations, whereas at least one other, large study found no association with event-free survival (EFS) or OS [38].

Mutant-to-wild-type allelic ratio, insertion site, ITD length, karyotype, and the presence of a mutation in the *NPM1* gene appear to further influence the prognostic utility of *FLT3*-ITD in patients with newly diagnosed *FLT3*-ITD-mutated AML. Higher allelic burden with the *FLT3*-ITD mutation has been specifically associated with worse outcomes in some studies but not in others [18, 39, 40]. For example, in a study evaluating the prognostic significance of *FLT3*-ITD in subgroups of patients with newly diagnosed *FLT3*-ITD-mutated AML, a threshold of mutant-to-wild-type ratio of >0.78 was significantly associated with shorter OS and disease-free survival (DFS) [18]. Similarly, in another study in patients with newly diagnosed *FLT3*-ITD-mutated AML, a high allelic ratio (≥0.51) and a *FLT3*-ITD insertion site in TKD1 predicted low complete remission (CR) rates and poor survival [39]. Notably, in both studies, patients were not treated with FLT3 inhibitors. In the RATIFY study, OS (not censored for transplant) was improved with midostaurin vs. placebo in groups with *FLT3*-ITD^high^ and *FLT3*-ITD^low^ allelic burdens, suggesting that both patients with high and low mutant-to-wild-type ratio may benefit from the addition of midostaurin [41]. Conversely, in a large cohort of patients in Medical Research Council trials, Linch et al. [40] found that the risk of relapse did not correlate with the allelic ratio. Taken together, the interpretation of the data on allelic ratio remains controversial in this setting, and further studies are needed to better elucidate the prognostic impact of allelic burden so that treatment decisions based on it may be optimized. Other *FLT3*-ITD-related variables that may have prognostic significance in this setting are the base pair size of the *FLT3*-ITD mutation and its insertion site; e.g., increasing *FLT3*-ITD size has been shown to be associated with decreasing OS and RFS [42–44].

## Prognostic impact of *FLT3* mutations in relapsed/refractory AML

An important concept in relapsed AML is that of clonal evolution, whereby mutations, such as *FLT3*-ITD mutations, that were not originally detectable at diagnosis can appear at relapse and may further affect prognosis [20, 21, 45–47]. In this setting (i.e., at relapse), AML is more oligoclonal, with leukemic clones harboring multiple adverse-risk genetic mutations, and appears to be more dependent on, or “addicted” to, FLT3 signaling, at least in vitro [48]. In most patients with a *FLT3*-ITD mutation at diagnosis, the *FLT3*-ITD mutation is retained at relapse, with a higher allelic burden at relapse than at diagnosis [48]. However, other clonal possibilities may occur as the disease progresses from diagnosis to relapse. Pooled data from several studies have shown that nearly 20% of patients with AML have a newly detectable or lose a previously detectable *FLT3*-ITD or *FLT3*-TKD mutation at relapse [46]. More specifically, *FLT3*-ITD mutations are newly detected at relapse more often than *FLT3*-TKD mutations (8% vs. 2%), whereas previously detected *FLT3*-TKD mutations are lost at relapse more frequently than *FLT3*-ITD mutations (7% vs. 4%) [46]. This pattern is consistent with observations suggesting that AML harboring a *FLT3*-TKD mutation at diagnosis may be more chemosensitive than AML harboring a *FLT3*-ITD mutation [38, 46]. Although the mechanisms underlying alterations in *FLT3* mutational status during clonal evolution remain unclear, it has been hypothesized that, in some cases, *FLT3* mutations may simply be present at diagnosis at levels below the limits of detection of conventional assays. Therefore, clones with *FLT3*-ITD mutations, although undetectable at diagnosis, may eventually become dominant at relapse due to the survival advantage conferred by the mutation. This is especially true after the selective pressure of harsh chemotherapy.

Clonal evolution is particularly important because gaining *FLT3*-ITD mutations at relapse has been associated with shorter OS than maintaining WT *FLT3* mutations [49]. *FLT3*-ITD mutations at relapse have also been shown to be an independent negative prognostic factor in patients in whom induction chemotherapy failed [50]. Moreover, Wattad and colleagues showed that patients with *FLT3*-ITD-mutated AML on salvage therapy had a high risk of relapse even after a potentially curative allogeneic hematopoietic stem cell transplant (alloHSCT), with the *FLT3*-ITD allelic ratio correlating directly with survival [50]—an observation that underscores patients’ poor prognosis in this setting. Similar findings were observed in another study evaluating the effect of pretreatment characteristics on clinical outcomes, whereby patients with *FLT3*-ITD at relapse had a very low probability of achieving a second CR with standard intensive therapy and patients with a high *FLT3*-ITD allelic ratio continued to have a dismal prognosis even after alloHSCT [51]. Overall, as in the newly diagnosed treatment setting, the presence of a *FLT3*-ITD mutation in relapsed/refractory AML is associated with shorter duration of remission, increased risk of relapse, and decreased OS following standard-of-care therapy.

As the use of molecular data for predicting prognosis in AML becomes more common, one application of these data may be to define the role of HSCT in various prognostic groups. For example, Oran et al. recently showed that alloHSCT at first CR is associated with a prolonged RFS and OS that is independent of the *FLT3*-ITD allelic ratio and *NPM1* mutation status in patients with a *FLT3*-ITD mutation [52]. Similarly, Ho et al. identified a clear EFS and OS benefit for alloHSCT vs. consolidation chemotherapy in patients identified as having high-risk *FLT3*-ITD-mutated AML and in patients with low-risk *FLT3*-ITD-mutated AML and WT *NPM1* who received alloHCT in first remission; in patients with low-risk *FLT3*-ITD-mutated AML and *NPM1* mutation a benefit from alloHSCT was not evident in terms of EFS and OS [53]. While some might interpret such studies to indicate that some subsets of patients with *FLT3*-ITD-mutated AML (defined by *NPM1* mutation status and low allelic ratio) should receive consolidation chemotherapy in preference to alloHSCT, the lack of a standard method of determining the allelic ratio makes this problematic. Given that no studies we are aware of have found alloHSCT to be harmful in these patients, a safer course is simply to offer transplant to all patients with *FLT3*-ITD-mutated AML in first remission when feasible.

Of note, the clinical course of AML is also influenced by specific combinations of mutations rather than individual mutations, as demonstrated in a recent study assessing the driver landscape in AML through the identification of nonoverlapping subgroups of patients [24]. The design of this study allowed a full genomic classification and found, for example, that the *NPM1* mutation carries a favorable prognosis only in the absence of a *FLT3*-ITD mutation (or *FLT3*-ITD with a low allelic ratio), whereas mutations in both *ASXL1* and *RUNX1* confer a poor prognosis, especially when they co-occur [54]. Collectively, although clinical outcomes in *FLT3*-ITD-mutated AML are complex and impacted by multiple factors, such as the patient’s baseline characteristics and co-occurring mutations, there is robust evidence that *FLT3*-ITD is an important prognostic biomarker, as recognized in international guidelines (e.g., NCCN and ELN).

## FLT3 inhibitors

**Fig. 1:**
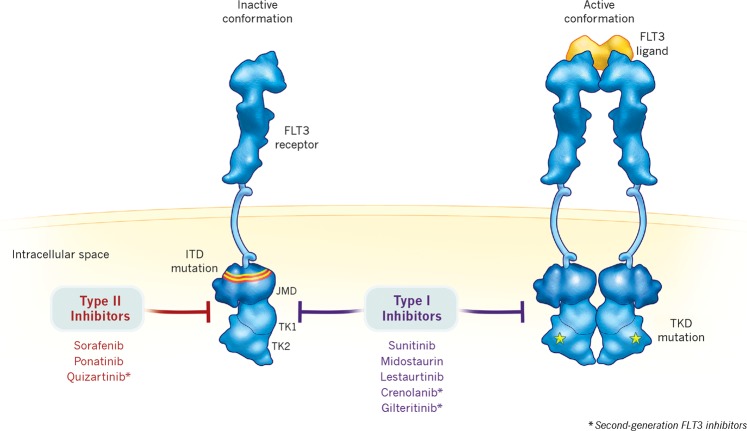
Type I FLT3: inhibitors bind the FLT3 receptor in the active conformation, either near the activation loop or the ATP-binding pocket, and are active against ITD and TKD mutations. Type II FLT3 inhibitors bind the FLT3 receptor in the inactive conformation in a region adjacent to the ATP-binding domain. As a result of this binding affinity, type II FLT3 inhibitors prevent activity of ITD mutations but do not target TKD mutations [81]. FLT3, FMS-like tyrosine kinase; ITD, internal tandem duplication; JMD, juxtamembrane domain; TK, tyrosine kinase; TKD, tyrosine kinase domain

Given the high frequency with which *FLT3* mutations occur in AML, a number of TKIs are under development that disrupt the oncogenic signaling initiated by FLT3. In addition to a variety of improved treatment strategies in AML, the recognition that *FLT3*-ITD is an adverse prognostic marker, the integration of FLT3 inhibitors into the treatment algorithm, and the increased use of alloHSCT have led to improvements over the past 15 years in clinical outcomes in patients with *FLT3*-ITD-mutated AML. Notably, this trend was observed retrospectively in a single-tertiary-center study evaluating differences in clinical outcomes in patients with newly diagnosed *FLT3*-ITD-mutated AML from 2000 to 2014, whereby a higher proportion of patients achieved CR in successive years and the corresponding median OS and median time to relapse increased significantly and incrementally over time with the introduction of alloHSCT and FLT3 inhibitors for the treatment of patients with *FLT3*-ITD mutations [55]. Overall, the use of FLT3 inhibitors, compared with historical outcomes prior to their emergence, has demonstrated a substantial clinical benefit in the relapsed/refractory AML setting and offers promising treatment strategies for patients with few options. For example, quizartinib, an oral, highly potent, and selective next-generation FLT3 inhibitor [56], significantly improved OS in a retrospective analysis in patients with *FLT3*-ITD-mutated AML who had relapsed after alloHSCT or after failure of second-line salvage chemotherapy compared with similar patients not treated with FLT3 inhibitors in the UK National Cancer Research Institute AML database (1988–2013) [57]. A summary of the first- and next-generation FLT3 inhibitors is presented in Table 4 and Fig. 1.

**Table 4:** First- and next-generation FLT3 inhibitors [79, 81, 88, 99, 100]

	Key pathways targeted (in addition to FLT3)	Developmental phase	Main toxicities
*First-generation FLT3 inhibitors*			
Sunitinib	VEGFR2, PDGFRβ, KIT, RET	Phase 2	Decreased appetite, headache, GI symptoms
Sorafenib	RAF, VEGFR1/2/3, PDGFRβ, KIT, RET	Phase 3	Skin rash, fatigue, diarrhea
Midostaurin	PKC, SYK, FLK-1, AKT, PKA, KIT, FGR, SRC, PDGFRα/β, VEGFR1/2	Approved for the treatment of newly diagnosed *FLT3*-mutated AML in combination with chemotherapy	Fever, flu-like symptoms, mouth sores, unusual bleeding or bruising
Lestaurtinib	JAK2/3, TrkA/B/C	Phase 2	Infections, sepsis, myocardial infarction
Ponatinib	LYN, ABL, PDGFRα, VEGFR2, FGFR1, SRC, KIT, TEK, RET	Phase 2	Pancreatitis
Tandutinib	KIT, PDGFRβ	Withdrawn	Muscle weakness
KW-2449	ABL, aurora kinase	Withdrawn	NA
*Next-generation FLT3 inhibitors*			
Crenolanib	PDGFRβ	Phase 3	Nausea, vomiting, transaminitis, fluid retention
Quizartinib	KIT, PDGFR	Phase 3	QTcF prolongation (especially at higher doses)
Gilteritinib	LTK, ALK, AXL	Phase 3	Diarrhea, fatigue, high liver function tests

*FGFR* fibroblast growth factor receptor, *FLT3* FMS-like tyrosine kinase 3, *GI* gastrointestinal, *JAK* Janus kinase, *NA* not applicable, *PDGFR* platelet-derived growth factor receptor, *PK* protein kinase, *VEGFR* vascular endothelial growth factor receptor

### First-generation TKIs

Several multitargeted TKIs, such as lestaurtinib, sunitinib, sorafenib, and midostaurin, have activity against FLT3 and have been investigated in patients with *FLT3*-ITD-mutated AML [31, 58–61]. Early-phase studies evaluating these first-generation FLT3 inhibitors as monotherapy generally demonstrated limited antileukemic activity, mixed results when these agents were combined with chemotherapy, and increased toxicity in some cases [61, 62]. For example, lestaurtinib following salvage chemotherapy elicited no improvement in response rates or OS compared with salvage chemotherapy alone in patients with relapsed *FLT3*-mutated AML [63], and combining lestaurtinib with intensive chemotherapy yielded no overall clinical benefit in patients with newly diagnosed *FLT3*-ITD-mutated AML [64]. Limited single-agent antileukemic activity was also shown in the phase 1 study of sunitinib, in which treatment of patients with refractory or resistant AML elicited only short-lived, partial responses [65]. Conversely, a phase 1/2 study evaluating sunitinib in combination with standard induction and cytarabine/daunorubicin consolidation therapy demonstrated that 50% of patients with *FLT3*-ITD mutations and 38% with *FLT3*-TKD mutations achieved CR [61]. In this study, two patients receiving sunitinib 25 mg daily continuously from day 1 onward (dose 1) and one patient receiving sunitinib 25 mg daily only on days 1–7 of each cycle (dose −1) experienced dose-limiting toxicities (i.e., prolonged time to recovery of peripheral blood counts and hand–foot syndrome at dose 1 and neutropenia at dose −1) that necessitated dose reductions [61].

As for sorafenib, similarly limited single-agent antileukemic activity was observed in a phase 1 study evaluating sorafenib monotherapy in patients with relapsed/refractory *FLT3-*mutated AML (the study included two patients with other acute leukemias and a minority of patients without *FLT3* mutations [22%]), in which only 10% of patients achieved CR or CR with incomplete platelet recovery [32]. Conversely, a phase 2 trial in younger patients (aged ≤ 60 years) with previously untreated AML found that the addition of sorafenib to standard-of-care chemotherapy significantly prolonged EFS and RFS but not OS compared with placebo. However, this treatment regimen was also associated with increased toxicity [62]. Moreover, in an exploratory subgroup analysis, no EFS benefit was observed with sorafenib in the small group of patients with *FLT3*-ITD mutations; however, patients with non-*FLT3* mutations had significantly improved EFS and RFS [62]. In another study, the combination of sorafenib with standard 7 + 3 chemotherapy in elderly patients (aged > 60 years) with AML did not significantly improve survival [66], with similar results seen in a subgroup analysis of patients with *FLT3*-ITD-mutated AML [66]. Additionally, elderly patients in the sorafenib arm had a higher incidence of early death, primarily due to infections [66]. However, in a nonrandomized study of patients with previously untreated AML, the combination of sorafenib, cytarabine, and idarubicin resulted in high response rates at a median follow-up of 52 months. CR/CR with incomplete platelet recovery rates of 95% were achieved in patients with *FLT3*-ITD mutations. DFS and OS of 13.8 and 29 months, respectively, were also improved compared with historical outcomes in these patients [34]. As yet another example, encouraging response rates (46% overall response rate [ORR]) were achieved in a nonrandomized study of azacytidine plus sorafenib in patients with relapsed/refractory AML, including 93% with *FLT3*-ITD mutations [67]. Finally, in the post-alloHSCT setting, phase 1 data suggest that sorafenib has good tolerability at twice-daily doses between 200 and 400 mg and a 1-year progression-free survival of 85% in all patients was reported, including several patients who underwent transplant beyond their first CR [68].

The use of single-agent midostaurin in patients with relapsed/refractory *FLT3*-mutated AML was similarly shown to have limited antileukemic activity, although it was generally well tolerated [31, 69]. Therefore, midostaurin was further investigated in combination with standard 7 + 3 chemotherapy in patients with newly diagnosed AML (aged 18–59 years) with *FLT3* mutations and was shown to significantly improve EFS (hazard ratio = 0.78; *P* = 0.002) and OS (hazard ratio = 0.78; *P* = 0.009) compared with 7 + 3 chemotherapy alone [70]. On the basis of the outcomes from this pivotal phase 3 trial (RATIFY), midostaurin (in combination with standard cytarabine and daunorubicin induction and cytarabine consolidation therapy) was approved by the US Food and Drug Administration (FDA) in April 2017 for the treatment of adult patients with newly diagnosed *FLT3*-mutated AML as detected by an FDA-approved test [71, 72]. In Europe, marketing authorization for midostaurin was granted by the European Commission on September 20, 2017, and includes an indication for single-agent maintenance therapy for adult patients in CR following induction/consolidation with 7 + 3 chemotherapy and midostaurin [73]. Despite that patients >60 years were not enrolled in RATIFY, there are no restrictions for midostaurin use in patients aged 65 and over, other than caution based on a patient's eligibility for concomitant chemotherapy and potential for comorbidities (i.e., greater frequency of concomitant disease or other drug therapy) [74]. Importantly, in patients receiving an HSCT, midostaurin should be discontinued prior to HSCT. Midostaurin was not approved by the FDA as maintenance therapy beyond induction and consolidation [71].

Data from the RATIFY trial showed that midostaurin consistently improved OS in all *FLT3* mutation subtypes (i.e., TKD, ITD low allelic ratio, and ITD high allelic ratio), with some differential effects observed among the four major genotypes studied (i.e., a significant OS and EFS benefit with midostaurin was observed only in the *NPM1-*WT/*FLT3*-ITD-high subgroup) and by gender (i.e., no significant OS benefit in men with *FLT3*-TKD mutations or women with *FLT3*-ITD mutations) [70, 75]. These results suggest that the prognostic impact and predictive value of individual mutations may be significantly impacted by concurrent mutations. However, given that patients across all *FLT3* subtypes—regardless of *FLT3*-ITD allelic ratio—benefited and that midostaurin inhibits multiple kinases, it is possible that midostaurin**’**s favorable effects may not stem purely from FLT3 inhibition but could be attributable at least in part to inhibition of other oncogenic pathways. Of note, 23% of the study population in the RATIFY trial had a *FLT3*-TKD mutation, which is a significantly larger percentage than the previously reported incidence of TKD mutations in the general AML population [41]. Patients with *FLT3*-TKD-mutated AML have lower white blood cell counts and generally have less-aggressive disease than patients with *FLT3*-ITD-mutated AML, allowing additional time for workup and screening for trials. This may have biased outcomes in this trial in favor of patients with a *FLT3*-TKD mutation, a phenomenon that was repeated in a similar trial of chemotherapy plus lestaurtinib [64]. Moreover, patients in the RATIFY trial were younger (median age = 47.9 years) than typical patients with newly diagnosed AML, highlighting the need for additional data for midostaurin in elderly fit and unfit patients [76, 77].

Although midostaurin is now approved, the debate is still ongoing as to how it impacts OS as well as its role in maintenance therapy. To this end, an exploratory analysis including CRs according to the protocol specifications (CRs up to day 60) showed that midostaurin improved the CR rate significantly after induction therapy when expanded CRs were counted (*P* *=* 0.04) [41]. Midostaurin was most effective in patients who received an alloHSCT in first CR, with a nonsignificant, in-trend better survival (*P* = 0.07) and a significantly lower cumulative incidence of relapse (*P* = 0.02) in all patients achieving a CR after induction therapy [70]. In contrast, patients who received chemotherapy without alloHSCT as consolidation therapy had a comparable cumulative incidence of relapse rate regardless of whether they received midostaurin. In a post hoc analysis, there appeared to be no benefit of midostaurin (DFS, *P* = 0.38; OS, *P* = 0.86) in patients who proceeded to midostaurin maintenance therapy [78].

Several phase 2 studies to further evaluate midostaurin in patients with newly diagnosed *FLT3*-ITD-mutated AML are ongoing. The first (AMLSG 16–10), a single-arm, phase 2 trial (NCT01477606; ongoing, but closed for recruitment) [79], is assessing the addition of midostaurin to chemotherapy during induction and consolidation as well as single-agent midostaurin maintenance for a maximum follow-up of 1 year in patients aged 18 to 70 years with newly diagnosed *FLT3*-ITD-positive AML. The second (RADIUS) is an ongoing, randomized, open-label, phase 2 trial evaluating the addition of midostaurin to standard of care vs. standard of care alone in the post-transplant setting in patients with *FLT3*-mutated AML who underwent alloHSCT and have not relapsed (NCT01883362). Preliminary results from both phase 2 trials suggest that these approaches are feasible [76, 80]. Of note, following early observations that patients with *FLT3*-WT experienced blast reductions with midostaurin, a phase 3 study in *FLT3*-WT AML has recently initiated (NCT003512197) [69].

### Next-generation TKIs

Overall, these multitargeted TKIs lack specificity for the mutated *FLT3*-ITD, which may explain their transient antileukemic activity, particularly when used as monotherapy in patients with relapsed disease [69], and may contribute to adverse effects from inhibition of multiple other kinases. To overcome these hurdles, several next-generation FLT3 inhibitors are under clinical investigation in AML, including gilteritinib, crenolanib, and quizartinib. These next-generation inhibitors have greater specificity for FLT3 and higher potency (logarithmically lower half-maximal inhibitory concentration) than multitargeted TKIs [58]. Gilteritinib and crenolanib are type I inhibitors that target both the inactive and active conformational states of the FLT3 kinase domain, whereas quizartinib is a type II inhibitor that is specific for the inactive conformation [81].

Next-generation FLT3 inhibitors have shown promising single-agent antileukemic activity in early clinical trials. For example, in a first-in-human, phase 1/2, dose-escalation and -expansion trial examining the effect of gilteritinib monotherapy in 252 patients aged ≥ 18 years with relapsed/refractory AML, gilteritinib treatment elicited a 40% ORR (including composite CR [CRc] and partial remission [PR]) in efficacy-evaluable patients (*n* = 249). The CRc rate was 30%, with the majority of responders having CR with incomplete blood count recovery (CRi) [29]. Response rates were considerably lower in patients with WT *FLT3* (ORR, 12%; CRc, 9%), whereas patients with *FLT3* mutations had an ORR of 49% and a CRc rate of 37%. Response rates increased with higher doses of gilteritinib; patients receiving ≥80 mg/day of gilteritinib achieved an ORR of 52% and a CRc rate of 41%. The median duration of response, defined as the time from the date of first CRc or PR until the date of relapse, was 17 weeks. Interestingly, the CRc rate with gilteritinib was 26% in patients treated with prior FLT3 inhibitors [29].

Additionally, single-agent crenolanib has demonstrated activity in the relapsed/refractory setting in two phase 2 studies in patients with relapsed/refractory *FLT3*-mutated AML. In the first, a single-center, phase 2 trial in 38 patients, crenolanib elicited a CRi rate of 23% in FLT3 inhibitor-naive patients and a CRi rate of 5% in those previously treated with FLT3 inhibitors. The median OS in the two groups was 55 and 13 weeks, respectively [30]. Results from the second, larger phase 2 study (*N* = 69) revealed a CRi rate of 39% and a PR rate of 11% in patients with relapsed *FLT3*-mutated AML who had not received prior FLT3 inhibitors and a CRi + PR rate of 28% in patients who had received prior FLT3 inhibitors. The median OS was 33.4 weeks in patients with relapsed/refractory *FLT3*-mutated AML who were TKI naive; OS was longest in those with *FLT3*-ITD mutations (34 weeks) and patients aged <60 years (33.4 weeks) [26].

Lastly, the type II inhibitor quizartinib—an oral, highly potent, and selective next-generation FLT3 inhibitor [56, 82, 83]—has been evaluated in QuANTUM-R, a global, randomized, open-label, phase 3 study (NCT02039726) examining the effect of quizartinib monotherapy vs. salvage chemotherapy (randomized 2:1) on OS in 367 patients with *FLT3*-ITD-mutated AML who are refractory to or have relapsed after first-line therapy. Other than rare cases of grade ≥3 QTcF prolongation with quizartinib, adverse event (AE) rates were comparable between the two arms.

Quizartinib has also been shown to be highly active in phase 2 trials, resulting in a high proportion of responders across many patient types with relapsed/refractory disease [27, 28]. For example, in a large phase 2 study (two cohorts; 333 patients), single-agent quizartinib (90 or 135 mg/day) resulted in CRc rates of 46 to 56% and ORRs of 74–77%, improved OS in responders compared with nonresponders, and was generally well tolerated, with a manageable safety profile [27]. In patients aged ≥60 years with *FLT3*-ITD-mutated AML relapsed within 1 year of initial remission or refractory to first-line chemotherapy (cohort 1; *n* = 157), the CRc rate was 56% and the ORR was 77%. Median duration of CRc was 12.1 weeks in *FLT3*-ITD-positive patients and 16.4 weeks in *FLT3*-ITD-negative patients. Median OS was 25.4 and 19.1 weeks in *FLT3*-ITD-positive and *FLT3*-ITD-negative patients, respectively. Similarly, in patients aged ≥18 years with AML relapsed/refractory to second-line salvage chemotherapy or relapsed after HSCT (cohort 2; *n* = 176), the CRc rate was 46% and the ORR was 74%. The median duration of CRc was 10.6 weeks in *FLT3*-ITD-positive patients and 7.0 weeks in *FLT3*-ITD-negative patients. In this cohort, the median OS was 24.0 and 25.1 weeks in *FLT3*-ITD-positive and *FLT3*-ITD-negative patients, respectively. Toxicity was consistent with phase 1 data and was generally well managed by dose interruptions and/or reductions [27].

Consistent with observations from the large, two-cohort, phase 2 trial, a phase 2b study evaluating the effects of quizartinib at lower doses (starting doses of 30 or 60 mg/day) in a similar patient population (i.e., relapsed/refractory *FLT3*-ITD-mutated AML; *N* = 76) showed strong single-agent clinical activity, with an overall CRc of 47% [28]. The 60-mg/day dose (vs. 30 mg/day) was associated with a higher ORR (71%), median OS (27.3 weeks), and bridge to transplant rate (42%), reinforcing the promising antileukemic activity of single-agent quizartinib observed in earlier studies and warranting further investigation of the 60-mg dosing regimen [28]. Additionally, initial experience with quizartinib in combination with chemotherapy in younger [84] and older patients with newly diagnosed AML [85] and in combination with azacytidine or low-dose cytarabine in older patients with newly diagnosed *FLT3*-ITD-mutated AML and those with *FLT3*-ITD-mutated AML in first relapse (including myelodysplastic syndromes and chronic myelomonocytic leukemia) [86] indicated that the combinations were feasible and appeared effective in both younger and older patients. Quizartinib also seems to be well tolerated as a single-agent therapy following alloHSCT in patients with *FLT3*-ITD-mutated AML who are in remission [87]. Together, these findings suggest that targeting the *FLT3*-ITD driver mutation with a highly potent and selective FLT3 inhibitor is a promising clinical strategy to help improve clinical outcomes in patients with very few options.

From a safety perspective, AEs associated with next-generation FLT3 inhibitors are generally manageable. Common treatment-related AEs  with gilteritinib were diarrhea (37%), anemia (34%), fatigue (33%), increased aspartate aminotransferase (26%), and increased alanine aminotransferase (19%) [29]. Crenolanib was associated with grade 3 gastrointestinal toxicities and mild or moderate nausea/vomiting, transaminitis, and fluid retention [26, 30]. Treatment-related treatment-emergent AEs occurring in patients treated with higher doses of quizartinib (i.e., ≥90 mg/day) were primarily myelosuppression and grade ≥3 QTcF prolongation (10%), which was reversible and successfully managed by treatment interruptions or dose reductions. Lower doses of quizartinib were associated with significantly lower rates of QTcF prolongation (<5% grade ≥3 QTcF prolongation) while maintaining high levels of efficacy.

### Ongoing trials of FLT3 inhibitors

New TKIs, such as ponatinib and FLX925, are being evaluated in phase 1 studies [88, 89]. However, none of these agents has demonstrated appreciable single-agent activity thus far. Given the favorable response and safety profiles of next-generation FLT3 inhibitors, several phase 3 trials are currently underway with gilteritinib, crenolanib, and quizartinib in a variety of settings. Two phase 3 studies are evaluating the effects of gilteritinib as maintenance therapy following alloHSCT (NCT02997202) and following induction/consolidation therapy (NCT02927262), respectively. Crenolanib is being evaluated in two phase 3 studies, including in combination with chemotherapy in patients with relapsed/refractory AML and *FLT3* mutations (NCT02298166) and in a randomized, head-to-head study of crenolanib vs. midostaurin in combination with standard first-line treatment for AML (NCT03258931). In addition to QuANTUM-R (described above), quizartinib is being evaluated in QuANTUM-First, a global, randomized, double-blind, placebo-controlled, phase 3 study (NCT02668653) examining the effect of quizartinib plus standard induction and consolidation chemotherapy followed by single-agent quizartinib on EFS in patients with newly diagnosed primary *FLT3*-ITD-mutated AML. The results from these studies may provide additional treatment options to a particularly vulnerable patient population.

## Mechanisms of resistance to FLT3 inhibitors

Despite significant progress in the development of newer FLT3 inhibitors with greater potency and specificity, emergence of resistance poses a significant challenge [81]. Both inherent and acquired mechanisms contribute to drug resistance. WT FLT3 is sensitive to FLT3 ligand and is relatively resistant to FLT3 inhibitors; therefore, the presence of WT FLT3 in most patients with *FLT3*-ITD mutations may contribute to resistance to FLT3 inhibitors. High levels of FLT3 ligand found in the BM microenvironment during induction as well as consolidation therapy can lead to persistent activity of the FLT3/MAPK pathway and provide survival signals to leukemic blasts, even in the presence of FLT3 inhibitors at levels that produce effective inhibition of FLT3 kinase activity in vitro [90, 91]. Persistent activation of pathways downstream of FLT3, such as MAPK and STAT5, has also been shown to contribute to inherent resistance to FLT3 inhibitors [92, 93]. Suboptimal efficacy of FLT3 inhibitors similarly may arise due to inadequate drug concentrations in the plasma, possibly due to rapid metabolism in the liver by cytochrome P450 A4 (CYP3A4) enzymes [94]. Finally, microenvironmental factors may influence the sensitivity of leukemic cells to TKIs. Preclinical studies have demonstrated that CYP3A4 expressed in BM stromal cells enhances drug metabolization and contributes to BM microenvironment-mediated FLT3 TKI resistance [95]. Acquired *FLT3* point mutations such as TKD mutations may arise from therapy with type I and II FLT3 inhibitors and mediate resistance [25, 96].

Secondary mutation-driven acquired resistance represents a common but complex mechanism underlying resistance to targeted therapies. Different FLT3 kinase inhibitors generate distinct, nonoverlapping secondary *FLT3* resistance mutations [97]. Additional resistance mechanisms include acquisition of other gene mutations and activation of alternative signaling pathways during treatment with FLT3 inhibitors [92]. Alterations in key signaling pathways in FLT3 TKI-resistant cell lines and primary samples reveal other forms of resistance, including activation of PI3K/AKT and/or RAS/MEK/MAPK pathways as well as continued expression of genes involved in *FLT3*-mediated cellular transformation [92].

An important strategy to overcome resistance to chemotherapy in many tumor types has been the use of combination regimens [81]. To this end, several ongoing studies are evaluating the utility of combining different agents that inhibit key signaling pathways through different modes of action or by using two or more agents that target different leukemic cell survival signaling pathways. Planned and ongoing studies investigating this clinical strategy include combinations of FLT3 inhibitors with approved antileukemic therapies, such as hypomethylating agents, low-dose cytarabine, CPX-351, and investigational agents (e.g., MDM2, BCL-2, IDH1/2, bromodomain, MEK, and CYP3A4 inhibitors). Combination therapies not only may improve response rates but also may produce more durable remissions in patients with *FLT3*-mutated AML.
